# Prognostic value of SEC61G in lung adenocarcinoma: a comprehensive study based on bioinformatics and in vitro validation

**DOI:** 10.1186/s12885-021-08957-4

**Published:** 2021-11-13

**Authors:** Qunhao Zheng, Zhiping Wang, Mengyan Zhang, Yilin Yu, Rui Chen, Tianzhu Lu, Lingyun Liu, Jiayu Ma, Tianxiu Liu, Hongying Zheng, Hui Li, Jiancheng Li

**Affiliations:** 1 Fujian Medical University Cancer Hospital, Fujian Cancer Hospital, Fuzhou, 350014 China; 2 Jiangxi Cancer Hospital of Nanchang University, Nanchang, Jiangxi China

**Keywords:** SEC61G, Lung adenocarcinoma, Novel biomarkers, Prognosis, Proliferation, Invasion

## Abstract

**Background:**

Studies have shown that the Sec61 gamma subunit (SEC61G) is overexpressed in several tumors and could serve as a potential prognostic marker. However, the correlation between SEC61G and lung adenocarcinoma (LUAD) remains unclear. In the current study, we aimed to demonstrate the prognostic value and potential biological function of the SEC61G gene in LUAD.

**Methods:**

Public datasets were used for SEC61G expression analyses. The prognostic value of SEC61G in LUAD was investigated using the Kaplan–Meier survival and Cox analyses. The correlation between the methylation level of SEC61G and its mRNA expression was evaluated via cBioPortal. Additionally, MethSurv was used to determine the prognostic value of the SEC61G methylation levels in LUAD. Functional enrichment analysis was conducted to explore the potential mechanism of SEC61G. Also, single sample GSEA (ssGSEA) and TIMER online tool were applied to identify the correlation between SEC61G and immune filtration. Furthermore, cell functional experiments were conducted to verify the biological behavior of SEC61G in lung adenocarcinoma cells (LAC).

**Results:**

SEC61G was upregulated in pan-cancers, including LUAD. High SEC61G expression was significantly correlated with worse prognosis in LUAD patients. Multivariate analysis demonstrated that high SEC61G expression was an independent prognostic factor in the TCGA cohort. (HR = 1.760 95% CI: 1.297–2.388, *p* < 0.001). The methylation level of SEC61G negatively correlated with the SEC61G expression (R = − 0.290, *p* < 0.001), and patients with low SEC61G methylation had worse overall survival. (*p* = 0.0014). Proliferation-associated terms such as cell cycle and cell division were significantly enriched in GO and KEGG analysis. Vitro experiments demonstrated that knockdown of SEC61G resulted in decreased cell proliferation, invasion and facilitated apoptosis in LAC. GSEA analysis found that SEC61G expression was associated with the E2F targets. Moreover, SEC61G expression was negatively correlated with the immune cell infiltration including CD4+ T cell, CD8+ T cell, B cell, macrophage, neutrophil, and dendritic cell.

**Conclusion:**

Our study indicated that overexpression of SEC61G was significantly associated with poor prognosis of LUAD patients and the malignant phenotypes of LUAD cells, suggesting that it could be a novel prognostic biomarker and potential therapeutic target of LUAD.

**Supplementary Information:**

The online version contains supplementary material available at 10.1186/s12885-021-08957-4.

## Background

Lung cancer, one of the most common malignant tumors worldwide, remains the first leading cause of cancer deaths [1]. Lung adenocarcinoma (LUAD) is the most frequently diagnosed histological subtype of non-small cell lung cancer (NSCLC), followed by squamous cell carcinoma [2]. Various factors such as cigarette smoking, second-hand or passive smoking, air pollution, genetic alteration, asbestos, and radon put individuals under the risk of LUAD [3]. Although alternative treatments such as targeted therapy and immune therapy for LUAD patients have been progressed rapidly over the past decades, the average 5-year survival rate of patients with LUAD remains less than 20% [4, 5]. A better understanding of the biological processes and molecular mechanisms underlying lung cancer pathogenesis will be of great significance to clinicians and improve patients’ outcomes. Thus it is of vital importance to keep searching for new tumor biomarkers and other potential genetic targets [6].

SEC61G, also known as Sec61 gamma subunit, is a central member of the SEC61 complex, a heterotrimeric protein channel formed by three subunits, SEC61 α, β, and γ [7]. Sec61 combined Sec62 and Sec63 serves as core component of the protein translocation machinery in the endoplasmic reticulum (ER) membrane and participates in protein folding, post-translational modification, translocation and unfolded protein response (UPR), especially under conditions of ER stress such as hypoxia and glucose deprivation in the tumor microenvironment [8, 9]. High frequency of mutation and overexpression of SEC62 and SEC63 have been observed in kinds of cancers, suggesting the potential role of ER protein in tumor development [10, 11]. Several studies have demonstrated that SEC61G was overexpressed in Glioblastoma [12], gastric cancer [13], hepatocellular carcinoma [14, 15] and breast carcinomas [16]. SEC61G gene was also found to coamplify with epidermal growth factor receptor in patients with glioblastoma and served as a potential prognostic marker [12, 17]. However, the potential correlation between SEC61G and lung adenocarcinoma has not been characterized.

In this present study, we comprehensively investigated the prognostic impact of SEC61G expression in LUAD patients through the gene expression profile and the matching clinical information of LUAD patients from the Cancer Genome Atlas (TCGA) and the Gene Expression Omnibus (GEO) database. A nomogram based on several independent risk factors was constructed. Functional enrichment and Gene set enrichment analysis (GSEA) were performed to explore the underlying mechanism of SEC61G involved in LUAD pathogenesis. We also identified the association between SEC61G expression and genetic alteration and methylation. Additionally, single-sample Gene Set Enrichment Analysis (ssGSEA) and TIMER were used to explore the correlation between the infiltration of immune cells and the expression of SEC61G. Finally, cell functional experiments were performed to verify the biological behavior of SEC61G in lung adenocarcinoma cells (LAC). We hope our study will help others get further insight into understanding the potential role of SEC61G in tumor pathogenesis and contribute to the improvement of molecular targeted therapy and prognosis for LUAD patients.

## Methods

### Data acquisition

The gene expression data, phenotype data, and the corresponding clinicopathological information of the TCGA-LUAD project and other tumor were acquired from the UCSC Xena browser (version: 2019-07-20,http://xenabrowser.net/datapages/). Clinical parameters, including age, gender, TNM stage and pathological stage, were evaluated. Transcriptome profiling data of patients with LUAD in the GSE11969 dataset from the Gene Expression Omnibus (https://www/ncbi.nlm.nih.gov/geo/) database were used for external validation of survival analyses. The two databases’ exclusion criteria were as follows: cases without complete gene expression data and survival information. Finally, 497 cases were extracted for further analysis. Patients with LUAD were classified into low- and high-expression groups according to the optimal cut-off value of SEC61G.

The expression levels of SEC61G in various cancers including LUAD were acquired from the TIMER database [18]. (https://cistrome.shinyapps.io/timer/). The Broad Institute Cancer Cell Line Encyclopedia Database (CCLE; http://www.broadinstitute.org/ccle) was used to validate the expression levels of SEC61G in different types of cancer cell lines [19]. The datasets of IMvigor 210 Clinical Trial was also download via the IMvigor210CoreBiologies R package and used for the survival analyses [20].

### Co-expressed and functional enrichment analysis

We used R (version 3.6.1) to identify the genes co-expressed with SEC61G in the TCGA-LUAD cohort. Spearman’s correlation coefficient (*r*) was calculated to evaluate the correlation between SEC61G and co-expressed genes, of which |*r*| > 0.35 and *P* < 0.001 were selected. Meanwhile, co-expressed analysis was also performed to explore the correlation between SEC61G and ER-stress related genes which was obtained from the Molecular Signatures Database (https://www.gsea-msigdb.org/gsea/msigdb/genesets.jsp). To explore the underlying biological mechanism of SEC61G in LUAD pathogenesis, Metascape [21](https://metascape.org) was used conducted gene ontology (GO) analysis and Kyoto Encyclopedia of Genes and Genomes (KEGG) analysis for genes co-expressed with SEC61G as previously selected.

### Gene set enrichment analysis

Gene Set Enrichment Analysis (GSEA) is a computational method that determines whether a set of a priori defined genes show statistically significant and consistent differences between biological states [22]. In this study, We conducted GSEA using the “clusterProfiler” R package (3.8.0, [23]) to elucidate the statistically significant function and pathway difference between high and low SEC61G expression groups of LUAD. H.all.v7.0.symbols.gmt in the MSigDB Collections was used as the reference gene collection. The expression level of SEC61G was regarded as a phenotype label. Adjusted *P*-value < 0.001, FDR q-value < 0.001 and |NES| > 1.5 was considered as statistically significant.

### Analysis between SEC61G expression and its correlation with immune infiltration

We conducted the ssGSEA (single-sample Gene Set Enrichment Analysis) method from the GSVA package [24] in R software to investigate the correlation between SEC61G expression and the immune cell infiltration level based on the published signature gene lists [25]. Spearman correlation was carried out to evaluate the correlation between SEC61G and immune cell infiltration. TIMER online tool was applied to validate the association between the SEC61G expression and the level of immune cell infiltration.

### SEC61G methylation level and its prognostic analysis

The copy number variation (CNV) and methylation level data of SEC61G were acquired from the cBioPortal web platform (https://www.cbioportal.org/) and a comparison of the varying SEC61G gene expressions in SEC61G copy number variation groups (Kruskal-Wallis test) and the correlation between SEC61G methylation level and SEC61G gene expression (Spearman correlation) was conducted. The SMART online platform (http://www.bioinfo-zs.com/smartapp/) was used to visualize the methylation levels of SEC61G in pan-cancer and normal samples from the TCGA database. The UALCAN online tool (http://ualcan.path.uab.edu/) was used to identify the differences in the promoter methylation level of SEC61G between LUAD and normal tissues from TCGA data. MethSurv online tool (https://biit.cs.ut.ee/methsurv/) was used to explore the prognostic value of the SEC61G methylation level in the TCGA-LUAD cohort.

### Prognostic model generation and prediction

Univariate analysis and multivariate cox regression analysis were used to determine the optimal prognostic model. Then, a nomogram was constructed to predict the prognosis by R packages rms. The patients were stratified into high and low risk groups based on the optimal cut-off value. The difference in OS between the high-risk group and low-risk group was analyzed by the Kaplan-Meier method with a two-sided log-rank test. The concordance index(C-index) and calibration curves were used to evaluate nomogram models’ quality. The C-index is between 0.5 and 1.0, where 1.0 indicates the model has a perfect capacity to distinguish outcomes with the model correctly, and 0.5 indicates random probability. The calibration curve is evaluated graphically by plotting the nomogram’s predicted probability against the observed rates. Overlap with the reference line indicates that the consistency of the model is perfect.

### Cell lines and cell culture

Human lung carcinoma cell lines A549 and H1299 were obtained from ATCC. A549 cells were cultured in F12K medium supplemented with 10% foetal bovine serum (FBS, AusGeneX, Australia), 100 U/ml penicillin, and 100 mg/ml streptomycin. H1299 cells were cultured in RPMI 1640 medium supplemented with 10% FBS, 100 U/ml penicillin, and 100 mg/ml streptomycin. Cell cultures were maintained in a humidified incubator consisting 5% CO2 at 37 °C.

### Small interfering RNA (siRNA) transfection

Knockdown of the expression of SEC61G in lung cancer cells was accomplished by small interfering RNAs (siRNAs) transfection using the Lipofectamine™ 3000 transfection reagent, according to the manufacturer’s instructions. Three small interfering RNA (siRNAs) targeting SEC61G (si-1, si-2 and si-3) and siRNA negative control (NC) were obtained from GenePharma (D010003; Shanghai, China). siRNAs were designed and synthesized by GenePhama (Shanghai, China). The siRNA sequences were as follows:

- SEC61G si-1 sense: 5′-CAGCAAUAGGAUUUGCUAUAATT-3′;
- SEC61G si-1 antisense: 5′-UUAUAGCAAAUCCUAUUGCUGTT-3′;
- SEC61G si-2 sense: 5′-AUCUUAGAGAUUGGUGAACAATT-3′;
- SEC61G si-2 antisense: 5′-UUGUUCACCAAUCUCUAAGAUTT-3′;
- SEC61G si-3 sense: 5′-AGCCAAGUCGGCAGUUUGUAATT-3′;
- SEC61G si-3 antisense: 5′-UUACAAACUGCCGACUUGGCUTT-3′.

### Quantitative real-time polymerase chain reaction (qRT-PCR)

The Trizol RNA extraction kit was used to extracted total RNA from transfected cells. Then the reverse transcription kit was employed to reversely transcribe RNA into cDNA. Subsequently, qRT-PCR was conducted to measure the expression of SEC61G. The calculation of inhibitory efficacy of the genes was performed with 2 − ΔΔCt method. GAPDH acted as the internal references. The primer sequences are as follows:

- SEC61G-forward: 5′-ACGTGTCCCTGGCATTTTAG-3′;
- SEC61G-reverse: 5′-TCAGCCACCAACAATGATGT-3′;
- GAPDH-forward: 5′-GAAGGTGAAGGTCGGAGTC-3′;
- GAPDH-reverse: 5′-GAAGATGGTGATGGGATTTC-3′.

### Western blotting

RIPA extraction reagent (Beyotime, Shanghai, China) was used for lysing cells to extract the total protein, and BCA Protein Assay (Thermo-Fisher Scientific, Waltham, MA, USA) was utilized to determine the protein concentration. Subsequently, samples were subjected to SDS-PAGE. Following the electrophoresis, proteins were transferred to polyvinylidene difluoride (PVDF) membranes (Millipore, Billerica, MA, USA). Next, the membranes and primary antibody (anti-SEC61G: 1:1000, 111,472–2-AP, ProteinTech, USA; anti-β-actin: 1:100000, AC026, ABclonal, USA) were incubated overnight at 4 °C and then were incubated with secondary antibody (goat anti-rabbit IgG-HRP, 1:5000, AS014; ABclonal, USA) for 1 h at room temperature. Sequentially, protein bands were visualized using chemiluminescence.

### Cell counting kit-8 (CCK8) assay

After transfection, cells were seeded into a 96-well plate according to the standard of 5000 cells per well. The proliferation of A549 or H1299 cells in the si-SEC61G and NC group was detected respectively. Cell viability was measured every 12 h. Briefly, 10 μl CCK8 reagent (MCE, Shanghai, China) was added daily to each hole in the 96-well plates and incubated at 37 °C for 1.5 h. The microplate reader (Infinite® M1000 PRO, TECAN, Switzerland) was used to measure the OD value at 450 nm.

### Colony formation assay

Briefly, 1000 cells were seeded into a 6-well plate and incubated in culture medium with 10% foetal bovine serum at 37 °C. The plates were incubated at 37 °C, 5% CO2 incubator for 2 weeks with frequent observation. Then the supernatant was removed and cells were washed carefully twice with PBS. Cells were fixed with 4% paraformaldehyde for 0.5 h before dyeing with 0.1% crystal violet. The number of clones was counted directly by the naked eye. The size and number of clones were compared finally.

### Cell apoptosis

The culture medium was replaced with a serum-free medium after 48 h of transfection., Cells were obtained by centrifugation after another 24 h of starvation and then resuspended by cold PBS (4 °C) and then centrifuged again. The supernatant was carefully removed. Cells were spread around in the binding buffer and complied with the Annexin V-FITC staining manufacturer’s instructions (US Everbright® Inc., Suzhou, China). FACS Calibur flow cytometer (BD Biosciences, Mountain View, CA) was used to determine the effects of SEC61G on lung cancer cell apoptosis.

### Cell invasion assay

Cell invasion capability was conducted using the 24-well Transwell chambers (8 μm pore size; Corning, NY, USA). The transwell chambers were coated with 100 μl matrigel (5× dilution; 100 μL/well; BD Biosciences, Bedford, MA) in a 24-well plate at 37 °C for 4 h. Then the upper compartment containing 100 μL of serum-free medium was added with 2 × 104 cells. Meanwhile, the lower compartment was added with 600 μL of medium with 10% FBS. After being cultured for 48 h in a 37 °C incubator, cotton sticks were used to wipe off the cells remaining in the upper chamber, and the cells penetrating the filter were fixed with 4% paraformaldehyde and mixed with 0.4% crystal violet solution. Subsequently, the stained cells were observed and photographed under a microscope at 100× magnification (Olympus, Tokyo, Japan). At least four fields in each chamber were observed under the microscope. The average number of invasion cells in each microscopic fields was counted.

### Statistical analysis

All statistical analyses were performed with the statistical package IBM SPSS Statistics software (SPSS26.0), GraphPad Prism 8.0 and R (version 3.6.1). The Wilcoxon signed-rank test and Wilcoxon rank-sum test were used to analyze the expression of SEC61G in paired and non-paired samples. X-tile software (Version 3.6.1) was conducted to identify the optimal cut-off value. The relationships between clinicopathological features and SEC61G expression were evaluated using Wilcoxon signed-rank test and logistic regression. Univariate analysis and multivariate analysis were done by applying Cox logistic regression model to identify independent variables, including age, gender, T stage, N stage, M stage, pathological stage, and SEC61G expression. The 95% confidence interval (Cl) of HR was calculated to assess individual factors hazard risk. The “survival” R package (version:0.1.3) and the “survminer” R package (version 0.4.8) were used to draw survival curves. The DESeq2 package (version: 3.26.5) was used to identify differential expression analysis between low and high SEC61G expression groups. The student’s t-test was carried out for comparisons between each group in vitro experiments. All tests were two-sided, and a *P*-value < 0.05 was considered as statistically significant.

## Results

### SEC61G is overexpressed in lung adenocarcinoma

**Fig. 1:**
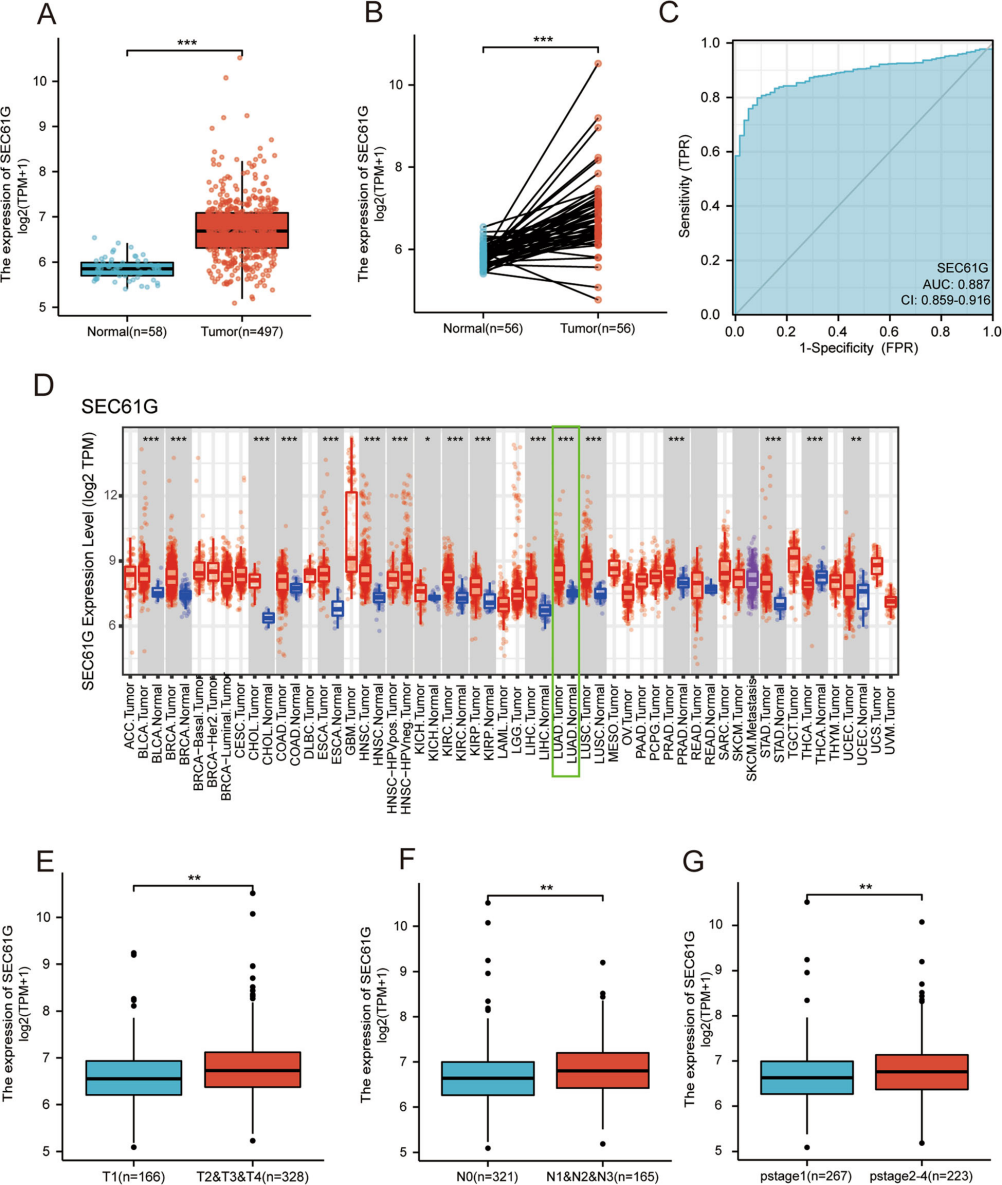
SEC61G expression in LUAD and other different types of human cancers from TCGA data. **A** Expression levels of SEC61G in LUAD and normal tissue; **B** The expression of SEC61G in LUAD and its paired adjacent tissues; **C** Receiver operating characteristic analysis (ROC) of SEC61G in LUAD; **D** The level of SEC61G expression in different tumor types from the TCGA database in TIMER. **E** The association of SEC61G expression and T classification in LUAD; **F** The association of SEC61G expression and N classification in LUAD; **G** The association of SEC61G expression and pathological stages in LUAD. (*P < 0.05, **P < 0.01, ***P < 0.001)

The results showed that SEC61G was highly expressed in lung adenocarcinoma tissue compared with normal tissue (*p* < 0.001) (Fig. 1A). In the paired specimens, SEC61G expression level in the LUAD group was significantly higher than that of the adjacent normal tissues (*p* < 0.001) (Fig. 1B). The ROC curve presented that the expression of SEC61G in LUAD was 0.887 (95% CI: 0.859–0.916) (Fig. 1C).

To further evaluate SEC61G expression in human cancers, we used TIMER to identify the SEC61G expression in multiple malignancies. The differential expression of SEC61G between the tumor and adjacent normal tissues is shown in Fig. 1D. SEC61G mRNA expression was significantly higher in bladder, breast, colorectal, esophageal, head and neck, kidney, liver, gastric, lung, prostate, gastric cancers and cholangiocarcinoma compared with the corresponding normal tissues (Fig. 1D). All tumor tissues presented in the TIMER database except the thyroid carcinoma showed higher SEC61G expression compared with the corresponding normal tissues. Then we investigated the expression levels of SEC61G in different cancer cell lines through CCLE database, as shown in the additional file 1.

As shown in Figs. 1E-G, increased SEC61G expression in LUAD was significantly associated with TN stage and pathological stage (T1 vs. T2/T3/T4 *P* < 0.01; N0 vs. N1/N2/N3 *P* < 0.01; pstage2&3&4 vs. pstage1 *P* < 0.01). These results indicated that LUAD with increased SEC61G expression was associated with a more advanced TN stage and pathological stage.

### High SEC61G mRNA expression correlates with adverse outcome in LUAD patients

**Fig. 2:**
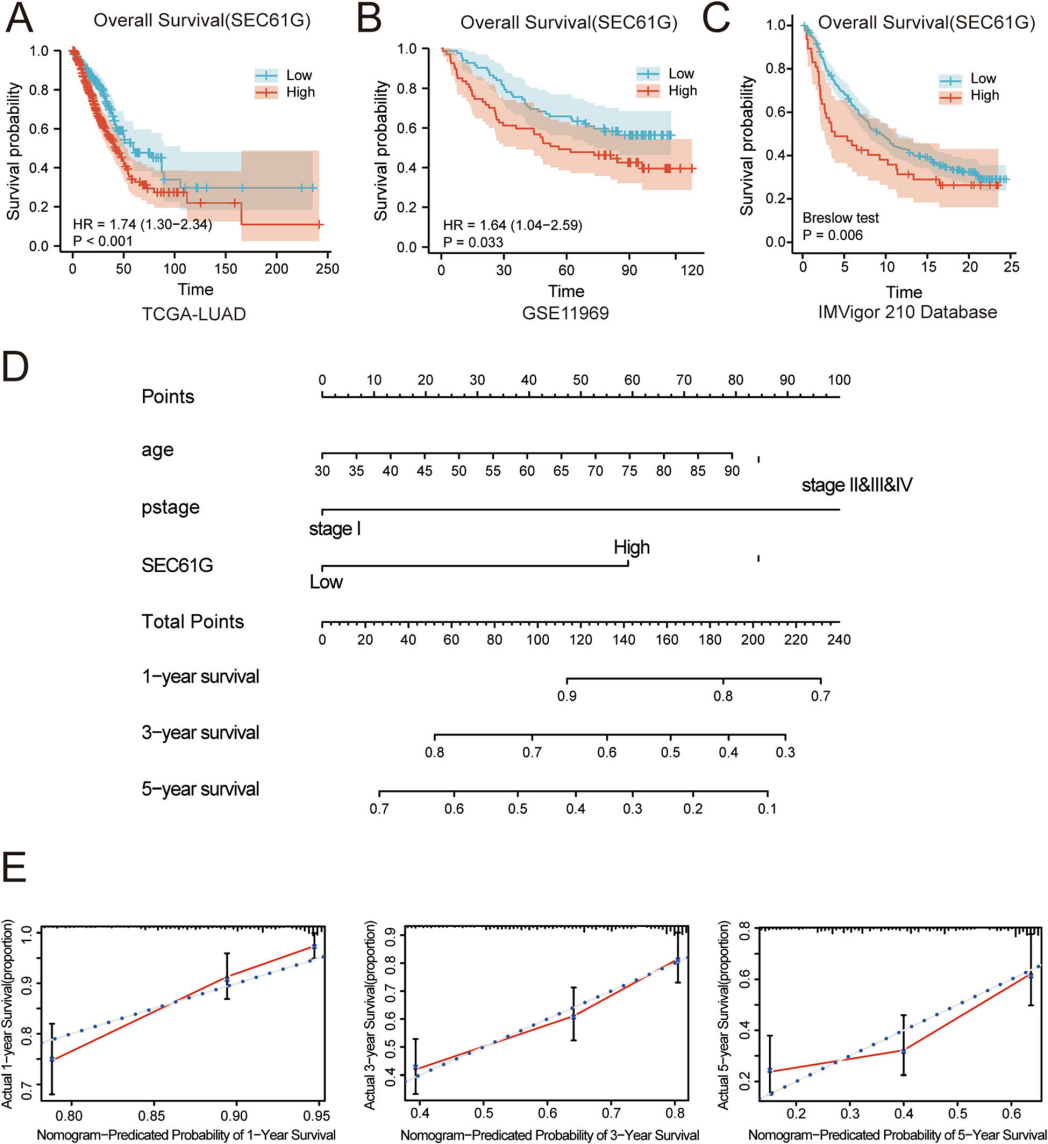
The prognostic value of SEC61G expression in LUAD. **A** Survival curve of OS from TCGA-LUAD data (*n* = 497); **B** Survival curve of OS from GSE11969 data (*n* = 149); **C** Survival curve of OS from IMVigor 210 data (*n* = 348); **D** A nomogram that integrates SEC61G and other prognostic factors in LUAD from TCGA data; **E** The calibration curves of the nomogram

We used the TCGA-LUAD cohort (*n* = 497) to investigate the correlation between SEC61G expression and LUAD patients’ prognosis. Survival analyses was also performed in the other tumor types whose SEC61G expression was differential expressed with normal tissue, as shown in additional file 2. Baseline characteristics of lung adenocarcinoma patients in the TCGA dataset were shown in Table 1. Kaplan–Meier analysis demonstrated that high SEC61G expression group is significantly related to shorter overall survival (OS) (HR=1.74 (1.30-2.34), *p* < 0.001) (Fig. 2A). We also used the GSE11969 datasets from the GEO database for validation, which was consistent with the result from the TCGA cohort (HR = 1.64 (1.04–2.59), *p* = 0.033) (Fig. 2B). Also, Univariate and multivariate Cox regression analysis was conducted to examine whether the SEC61G expression was an independent factor in LUAD patients. (Table 2) Finally, age, pathological stage, and SEC61G expression were identified as the independent prognostic factors. This indicated a role of SEC61G in the prognosis of LUAD. The above results demonstrated that SEC61G is a prognostic factor, and increased SEC61G expression level correlates with poor OS.

**Table 1:** Baseline characteristics of lung adenocarcinoma patient in the TCGA dataset

	TCGA (*N* = 497)
**Gender**	(%)
Male	228 (46.2%)
Female	269 (53.8%)
**Age at diagnosis**	(%)
< =70 years old	327 (67.1%)
> 70 years old	160 (32.9%)
**T stage**	(%)
T1	166 (33.6%)
T2&T3&T4	328 (66.4%)
**N stage**	
N0	321 (66.0%)
N1&N2&N3	165 (34.0%)
**M stage**	
M0	331 (93.2%)
M1	24 (6.8%)
**Pathological stage**	
stage1	267 (54.5%)
stage2&3&4	223 (45.5%)
**SEC61G expression**	
Low	273 (54.9%)
High	224 (45.1%)
**Vital status**	
Dead	180 (36.2%)
Alive	317 (63.8%)

**Table 2:** Univariate and multivariate Cox regression analyses of SEC61G expression for survival of LUAD patients in TCGA dataset

Characteristics	Total	Univariate analysis		Multivariate analysis	
	(N)	HR (95%CI)	*P* value	HR (95%CI)	*P* value
Gender (Male vs. Female)	497	0.954 (0.711 ~ 1.279)	0.752		
Age (>70y vs. <=70y) T stage	487	1.464 (1.081 ~ 1.982)	**0.014**	1.493 (1.096 ~ 2.035)	**0.011**
(T2–4 vs. T1)	494	1.678 (1.187 ~ 2.373)	**0.003**	1.257 (0.873 ~ 1.812)	0.188
N stage (N1–3 vs. N0)	486	2.637 (1.957 ~ 3.553)	**< 0.001**	1.414 (0.865 ~ 2.314)	0.167
M stage (M1 vs. M0)	355	2.129 (1.243 ~ 3.648)	**0.006**		
Pathologic stage (stage2–4 vs. stage1)	490	2.977 (2.184 ~ 4.058)	**< 0.001**	1.966 (1.164 ~ 3.319)	**0.011**
SEC61G (High vs. Low)	497	1.741 (1.296 ~ 2.388)	**< 0.001**	1.760 (1.297 ~ 2.388)	**< 0.001**

*HR* hazard ratio, *CI* confidence interval

### Development of predictive nomogram based on SEC61G and clinicopathological factors

A nomogram integrating those independent clinical risk factors (age, pathological stage and SEC61G expression) was constructed (Fig. 2C). A high total score predicted the low 1-, 3-and 5-year survival. And a low total score showed the opposite. The C-index for OS prediction was 0.696, with 1000 bootstrap resamples for the nomogram. And the calibration plots (Fig. 2D) showed good agreement compared with the ideal curves, indicating that our assembled nomogram has the stability for predicting LUAD patient prognosis in clinical practice.

### Hypomethylation associates with the expression of SEC61G and indicates a poor prognosis in LUAD

**Fig. 3:**
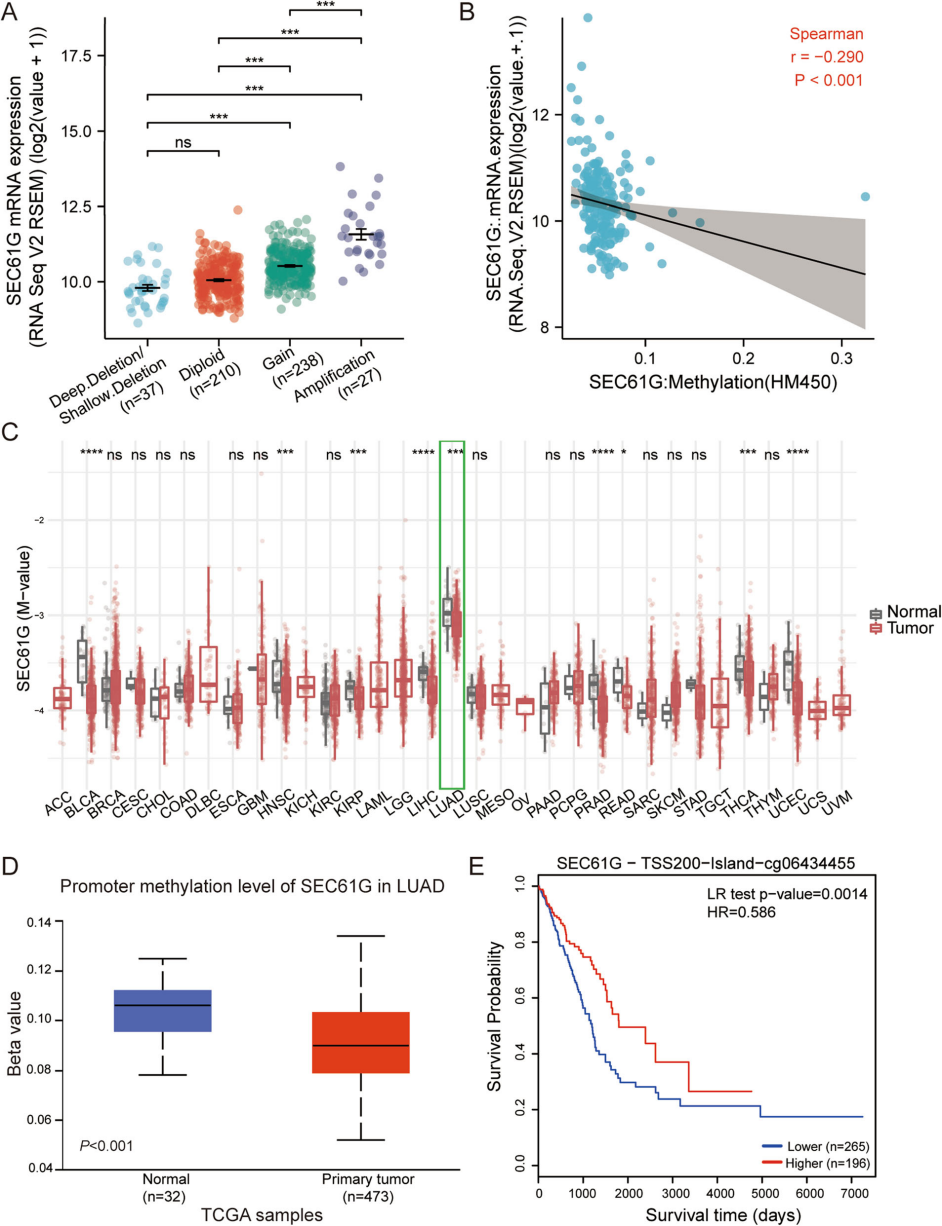
The copy number variation (CNV) and methylation of SEC61G in LUAD. **A** the expression level in different CNV of SEC61G; **B** the correlation between SEC61G methylation and its expression level; **C** The methylation levels of SEC61G in pan-cancer and normal tissues from TCGA data; **D** The promoter methylation of SEC61G in tumor tissues from TCGA-LUAD data; **E** the Kaplan-Meier survival of the promoter methylation of SEC61G in LUAD

The correlation between the mRNA expression of SEC61G and its copy number variation (CNV) was analyzed using cBioPortal. Patients with the amplification of CNV of SEC61G had a higher level of SEC61G expression in LUAD, but only 5.3% of patients (27/512) exhibited this (Fig. 3A). This indicated that CNV might not be the primary cause behind the high expressed SEC61G gene. We further investigated the association between SEC61G methylation and gene expression, and these results showed that SEC61G methylation negatively associated with SEC61G gene expression (R = − 0.290, *p* < 0.001) (Fig. 3B). Multiple malignancies showed a lower methylation level compared with normal tissues in the TCGA database, including lung adenocarcinoma (Fig. 3C). The UALCAN showed that the promoter methylation of SEC61G in lung adenocarcinoma tissues was significantly lower than that of adjacent normal tissues (*p* < 0.001) (Fig. 3D). In addition, the MethSurv online tool revealed that patients with lower SEC61G methylation had a worse overall survival. (*p* = 0.0014) (Fig. 3E). Furthermore, we also found that the expression of SEC61G was positively correlated with gene copy number and demethylation level in CCLE database, which was consistent with the results of our bioinformatics analysis, as shown in additional file 1.

### Functional enrichment analysis of SEC61G co-expressed genes

**Fig. 4:**
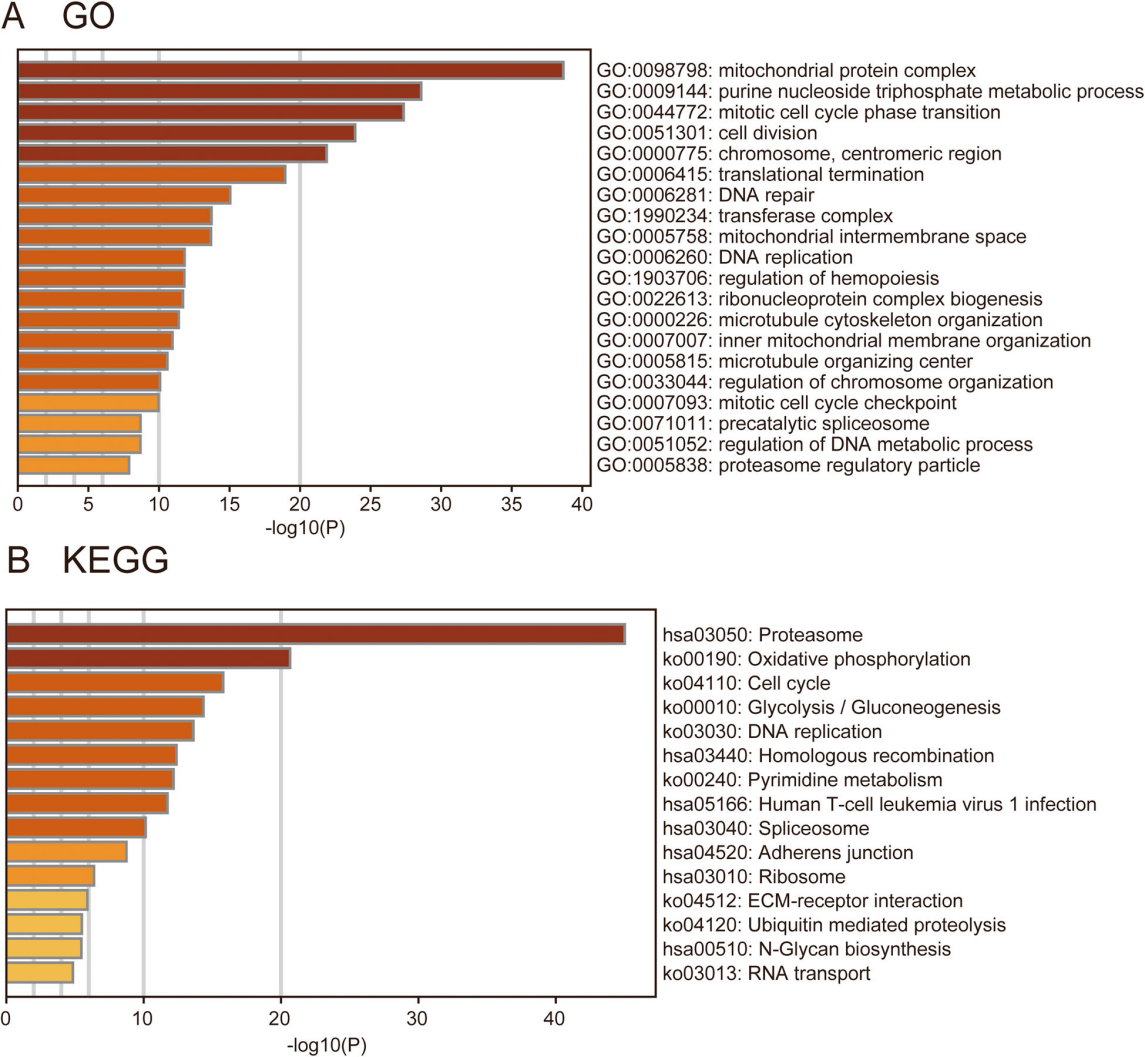
Functional enrichment of SEC61G co-expressed genes in LUAD. **A** Gene Ontology (GO) enrichment analysis; **B** Kyoto Encyclopedia of Genes and Genomes (KEGG) analysis

**Fig. 5:**
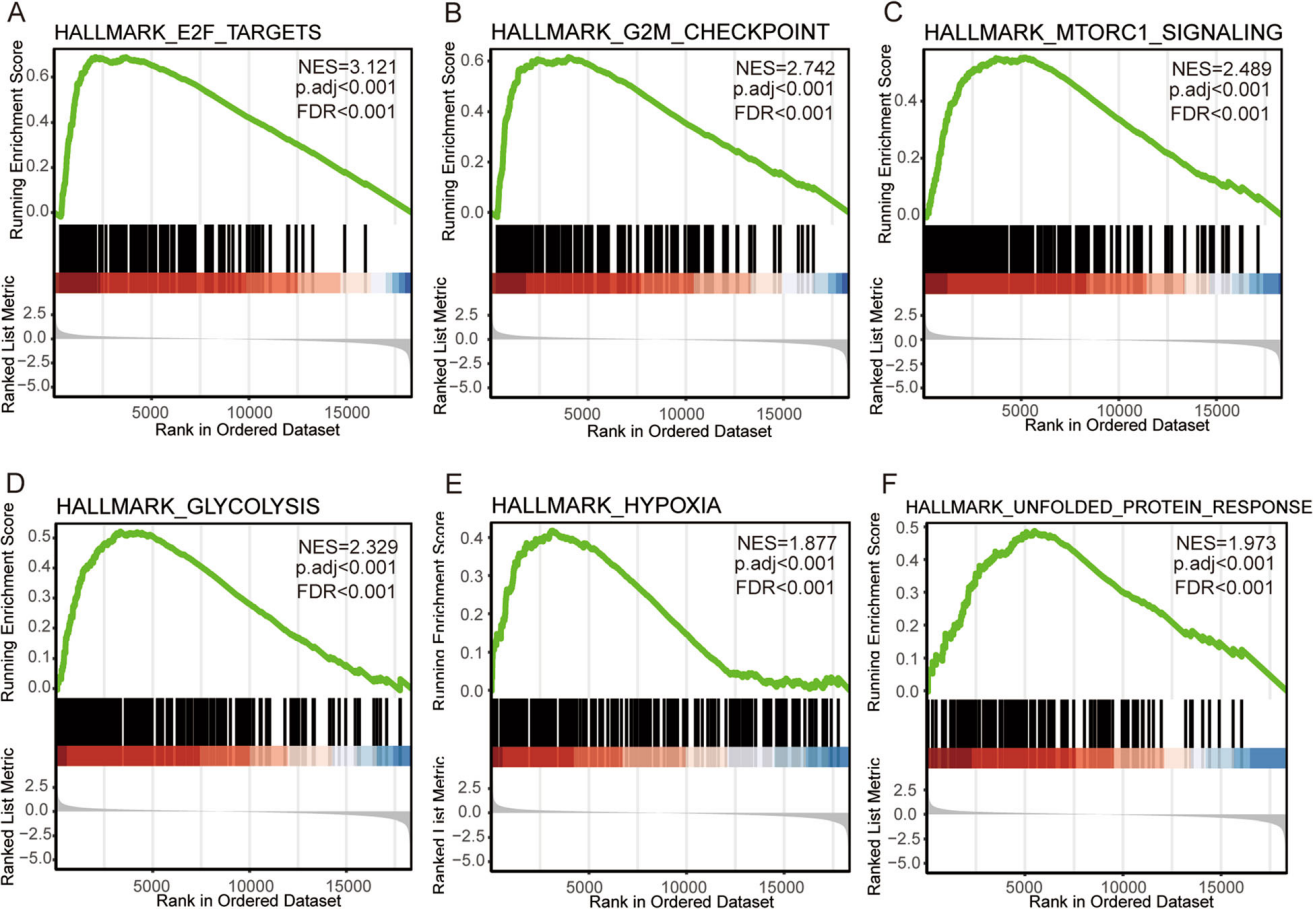
Enrichment plots acquired from the gene set enrichment analysis (GSEA). **A** Enrichment of genes in the E2F targets pathway by GSEA; **B** Enrichment of genes in the G2M checkpoint pathway by GSEA; **C** Enrichment of genes in the MTORC1 signaling pathway by GSEA; **D** Enrichment of genes in the glycolysis pathway by GSEA; **E** Enrichment of genes in the hypoxia pathway by GSEA; **F** Enrichment of genes in the unfolded protein response by GSEA

A total of 973 co-expression genes were identified, of which 560 genes were positively correlated and 413 were negatively correlated. Following this, the functions of co-expression in patients with LUAD were predicted using GO enrichment and KEGG pathway analysis. The top 10 GO enrichment items (Fig. 4A) were including mitochondrial protein complex, purine nucleoside triphosphate metabolic process, mitotic cell cycle phase transition, cell division, chromosome, centromeric region, translational termination, DNA repair, transferase complex, mitochondrial intermembrane space, DNA replication. KEGG pathway analysis (Fig. 4B) showed enrichment in the proteasome, cell cycle, oxidative phosphorylation, glycolysis/gluconeogenesis, DNA replication, Homologous recombination, Pyrimidine metabolism, Human T-cell leukemia virus one infection, spliceosome, adherens junction, ribosome, N-Glycan biosynthesis, ECM-receptor interaction, ubiquitin mediated proteolysis, and RNA transport pathways. We also performed a GSEA analysis to identify the potential pathways related to SEC61G. The most significantly enriched pathways were E2F targets, G2M checkpoint, glycolysis, MTORC1 signaling, MYC targets, oxidative phosphorylation, DNA repair, hypoxia, unfolded protein response. (Fig. 5) The ER-stress related genes with statistical differences in high and low SEC61G expression groups were shown in additional file 3.

### The correlation between SEC61G expression and immune cell infiltration

**Fig. 6:**
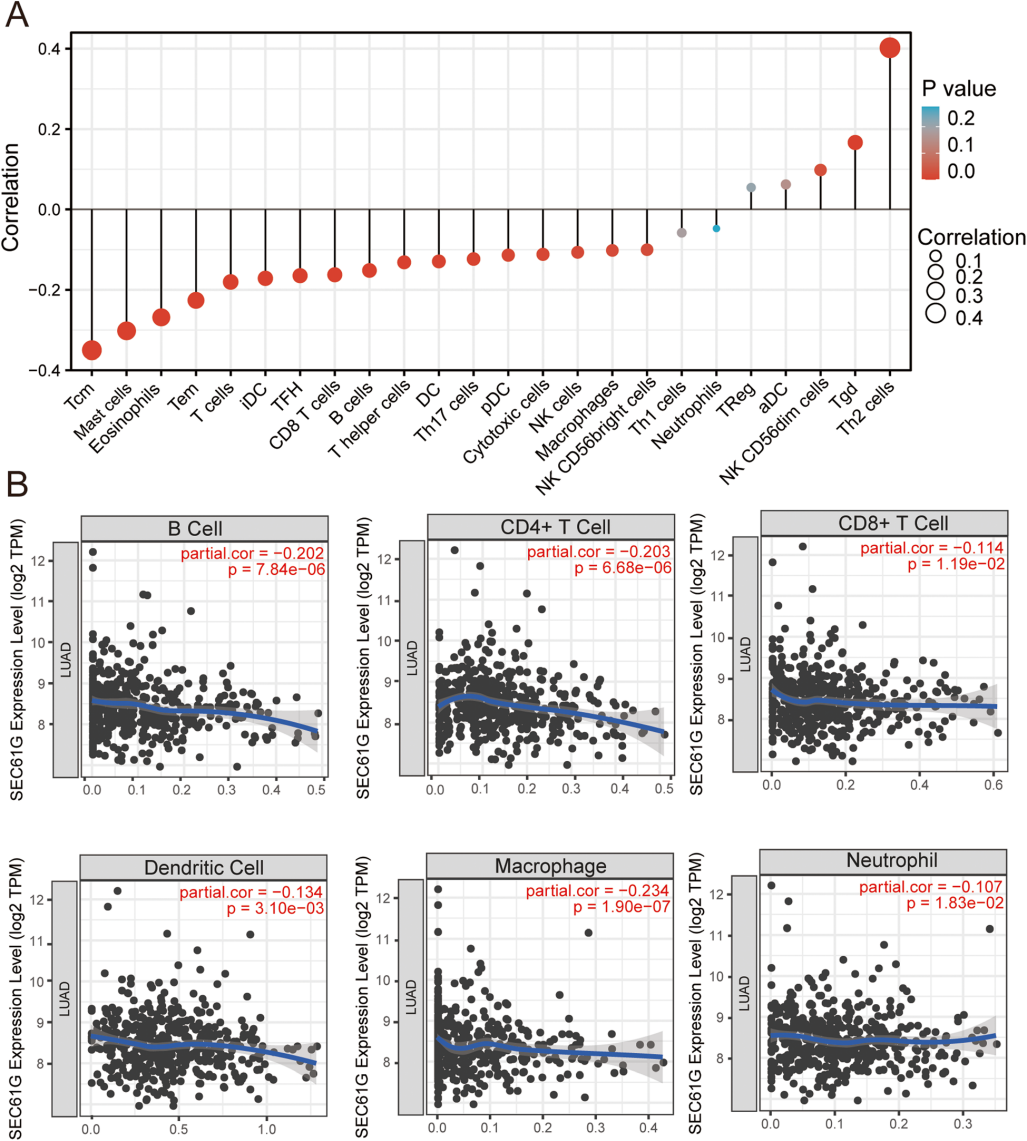
The correlation of SEC61G expression with immune infiltration level in LUAD. **A** The correlation of SEC61G expression with immune cell infiltration conducted by ssGSEA. **B** The correlation of SEC61G expression with immune cell infiltration in LUAD acquired from TIMER online tool

We further conducted ssGSEA to identify the potential relationship between the expression of SEC61G and immune cell infiltration. The correlation between immune cell infiltration and SEC61G expression is shown in Table 3 and Fig. 6A. The results showed that SEC61G expression positively correlated with the infiltration of Type 2 T helper cells, Gamma delta T cells, CD56 dim natural killer cells. On the contrary, Central memory CD4 T cells, Mast cells, Eosinophils, Effector memory CD8T cells, T cells, Immature dendritic cells, T follicular helper cells, CD8 T cells, B cells, T helper cells, Dendritic cells, type 17 T helper cells, Plasmacytoid dendritic cells, Cytotoxic cells, Natural killer T cells, Macrophages, CD56 bright natural killer cells were found to be negatively correlated with SEC61G expression. In contrast, SEC61G expression did not correlate with Activated dendritic cells, Type 1 T helper cells, Regulatory T cells, Neutrophils. Furthermore, the analyses by TIMER online tool showed that the level of SEC61G expression negatively correlated with the infiltration of B cells (R = − 0.202, *p* < 0.001), CD8+ T cells (R = − 0.114, *p* = 0.012), CD4+ T cells (R = − 0.203, *p* < 0.001), DCs (R = − 0.134, *p* = 0.003), Macrophage (R = − 0.234, *p* < 0.001) and Neutrophil (R = − 0.107, *p* = − 0.018)(Fig. 6B) What’s more, The IMvigor 210 study showed that metastatic urothelial cancer patients with higher SEC61G expression level had a better survival benefit from the atezolizumab immunotherapy than those patients with low SEC61G expression (*p* = 0.006, breslow test) (Fig. 2C).

**Table 3:** The association between the expression level of SEC61G and the immune infiltration in the tumor microenvironment

Immue cells	Spearman correlation	*P* value
Activated dendritic cells (aDC)	0.062	0.151
B cells	−0.152	< 0.001
CD8 T cells	−0.162	< 0.001
Cytotoxic cells	−0.112	0.010
Dendritic cells (DC)	−0.129	0.003
Eosinophils	−0.268	< 0.001
Immature dendritic cells (iDC)	−0.171	< 0.001
Macrophages	−0.102	0.019
Mast cells	−0.302	< 0.001
Neutrophils	−0.047	0.276
NK CD56bright cells	−0.100	0.021
NK CD56dim cells	0.098	0.023
NK cells	−0.106	0.014
Plasmacytoid dendritic cells (pDC)	−0.114	0.009
T cells	−0.180	< 0.001
T helper cells	−0.131	0.002
Central memory T cells (Tcm)	−0.350	< 0.001
Effector memeory CD4 T cells (Tem)	−0.226	< 0.001
T follicular helper cells (TFH)	−0.165	< 0.001
Gamma delta T cells (Tgd)	0.167	< 0.001
Th1 cells	−0.058	0.183
Th17 cells	−0.123	0.004
Th2 cells	0.402	< 0.001
Regulatory T cells	0.054	0.209

### Knockdown of SEC61G in LAC cell lines

**Fig. 7:**
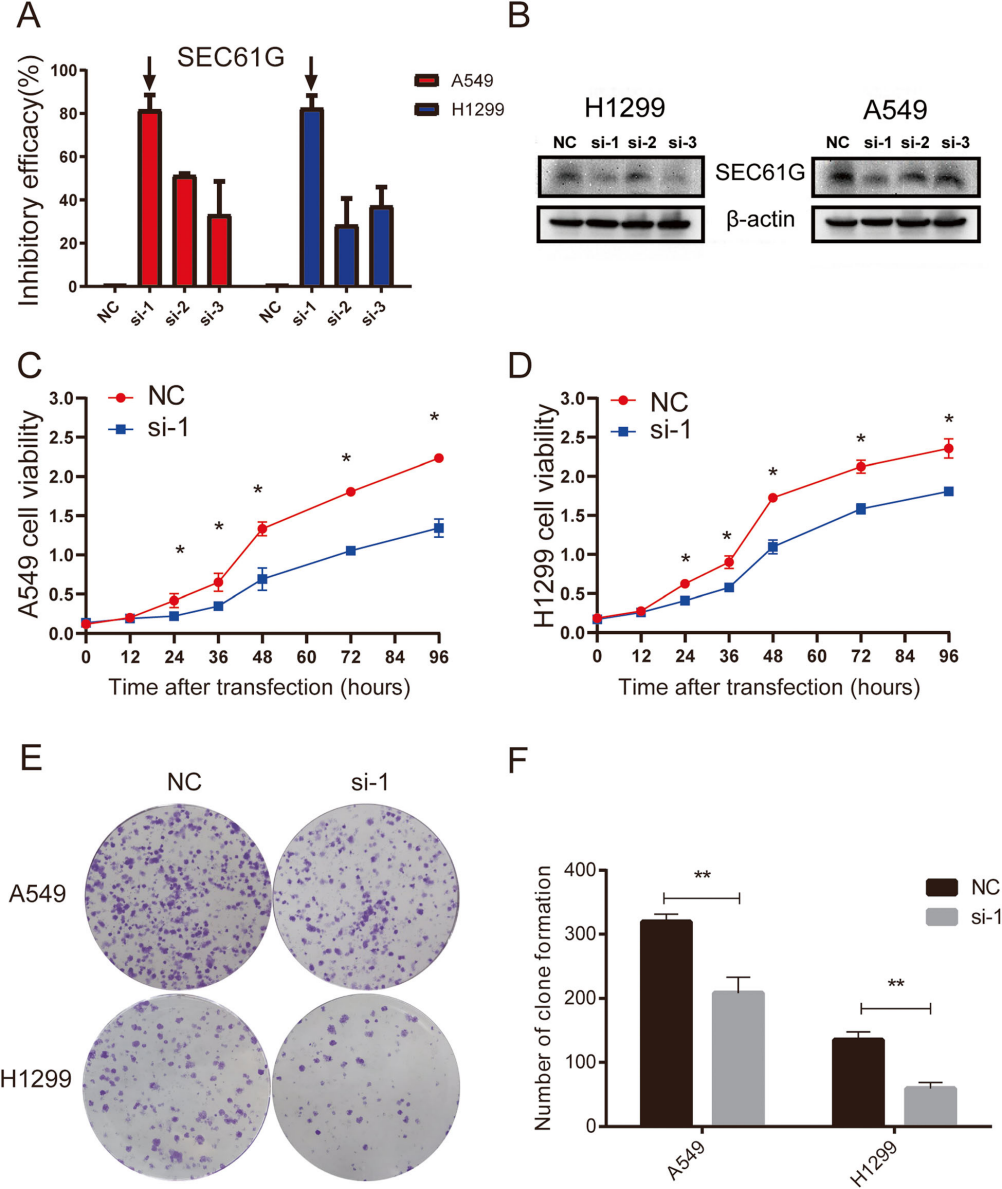
siRNA effect examination and cell proliferation capacity comparison. **A** qRT-PCR showed the inhibitory efficacy in A549 and H1299 cells transfected with si-1, si-2, si-3 and NC. **B** Western blotting showed the protein expression level of cells SEC61G in A549 and H1299 cells transfected with si-1, si-2, si-3 and NC. **C**-**D** CCK8 assay demonstrated the proliferation capacity of A549 and H1299 cells in si-1 group was significantly weaker than that of NC group. * P < 0.05 vs control. **E**-**F** Colony formation assay indicated that the clone number of A549 and H1299 cells transfected with si-1 was significantly less than that of NC group

The SEC61G expression was silenced by three small interfering RNAs; as a result, the qRT-PCR assay showed that si-1 had the best silencing effect among the three siRNAs against SEC61G in both A549 and H1299 cell lines (Fig. 7A). Then in western blotting assay, We found that the protein expression of SEC61G decreased significantly in the two cell lines transfected with si-1 compared to that in the same cell lines transfected with si-2, si-3 and NC (Fig. 7B). Taken together, these results demonstrate that si-1 was effective in inhibiting the expression of SEC61G, thereby si-1 was selected in subsequent experiments.

### Knockdown of SEC61G inhibited the malignant biological behaviors of LAC cells

**Fig. 8:**
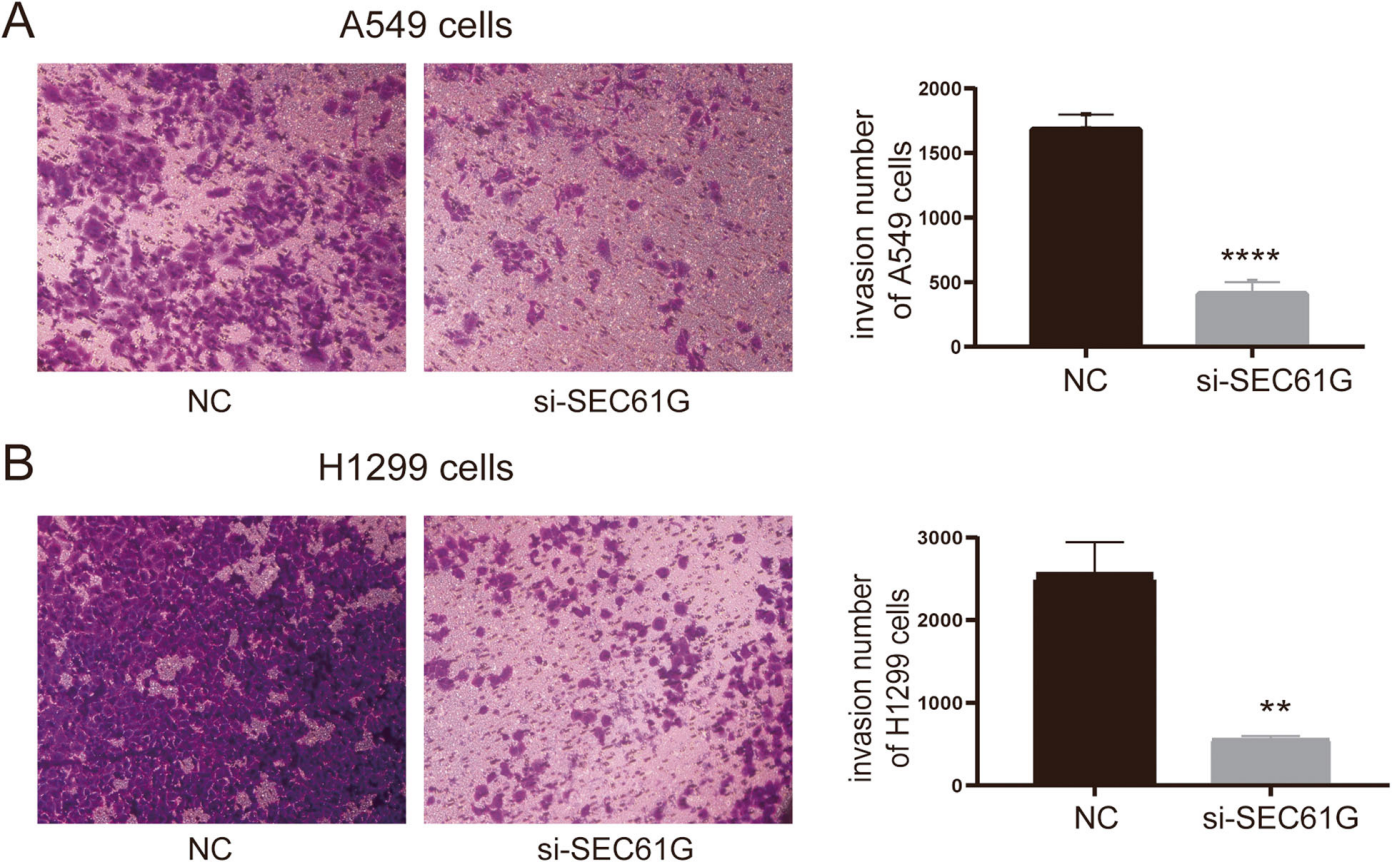
Knockdown of SEC61G inhibited cell invasion in lung adenocarcinoma cells. **A**-**B**. Invasion assay demonstrated that in comparison to those in NC group, cell invasion capacities were significantly reduced in the si-SEC61G groups. Data are expressed as mean ± standard deviation (SD). ** *p* < 0.01 and *** *p* < 0.001 compared with NC group

**Fig. 9:**
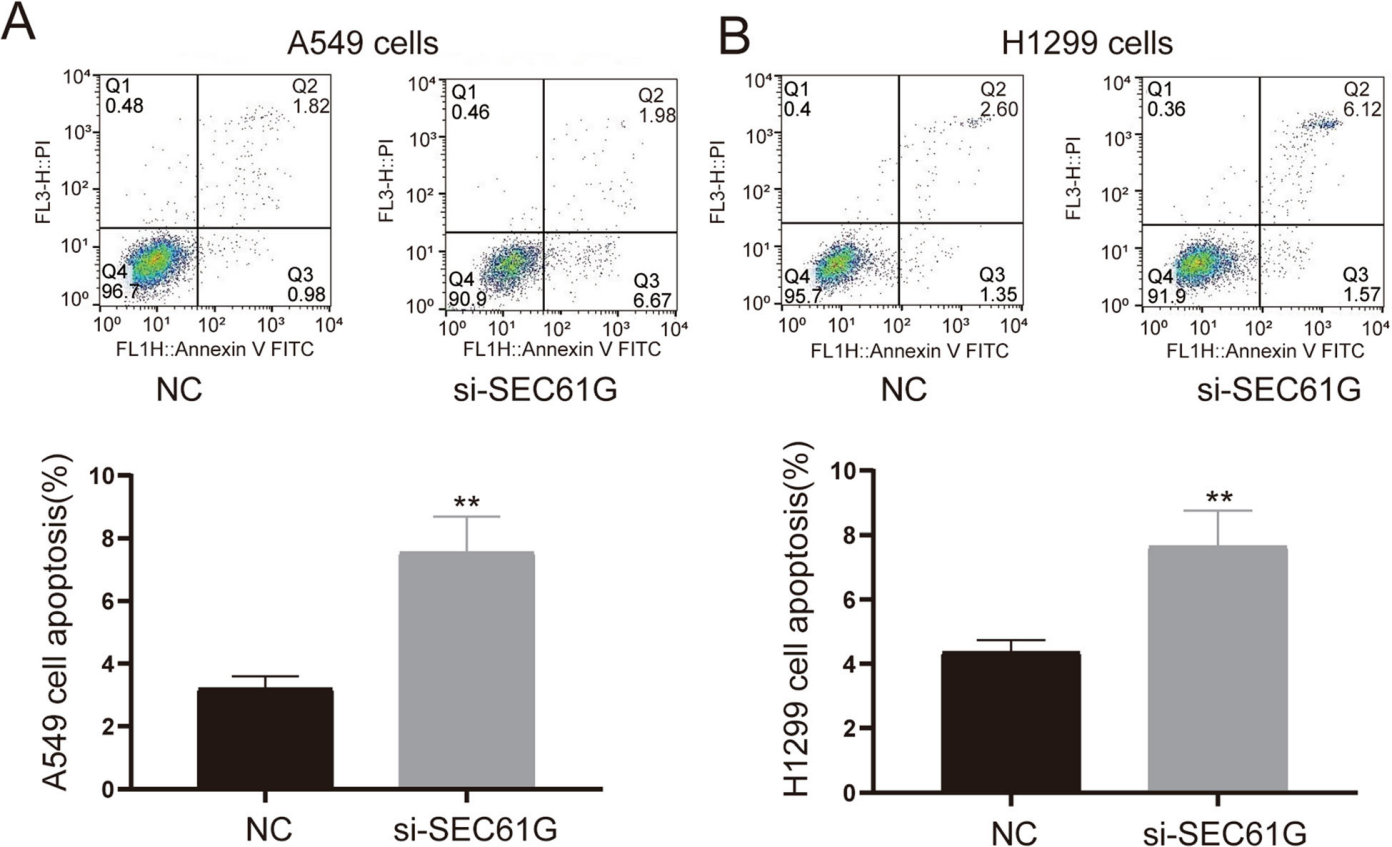
Knockdown of SEC61G enhanced cell apoptosis in lung adenocarcinoma cells. **A**-**B** PI-FITC-annexin assay demonstrated that knockdown of SEC61G significantly induced cell apoptosis. The statistic of apoptotic cells percentage in each group were quantified. Data are expressed as mean ± SD (n = 3). **p < 0.01; ****p < 0.0001

To explore the regulatory effects of SEC61G on LAC cells’ proliferation, apoptosis and invasion, a series of In vitro experiments were conducted. CCK-8 assays evinced that compared to the NC group, knockdown of SEC61G could significantly inhibit the multiplication of A549 and H1299 cells (Fig. 7C-D). Colony formation assay also showed that the number of clones of A549 cells and H1299 cells transfected with si-SEC61G was significantly less than that of the NC group (Fig. 7E-F). Transwell assay suggested that in comparison to those in the NC group, cell invasion capacities were significantly reduced in the si-SEC61G groups (Fig. 8A: t = 16.91, *P* < 0.0001; Fig. 8B: t = 8.469, *P* = 0.0011). PI-FITC-annexin assay demonstrated that knockdown of SEC61G significantly induced cell apoptosis, compared with the NC group. (Fig. 9A: t = 6.291, *P* = 0.0033; Fig. 9B: t = 4.901, *P* = 0.0080). These findings indicated that knockdown of SEC61G promoted apoptosis in lung adenocarcinoma cells, which could partly responsible for SEC61G depletion-induced cell proliferation suppression. The above evidence confirmed that knockdown of SEC61G could remarkably inhibit the malignant phenotypes of LAC cells.

## Discussion

Lung adenocarcinoma, which constitutes almost 50% of NSCLCs, is a severe global public health problem with high mortality and morbidity in cancer patients [1, 2]. Although newly molecular targeted therapy and immunotherapy have shed lights on NSCLC treatment, the therapeutic efficacy is still limited due to the high heterogeneity of lung cancer and lung cancer patients’ outcome is still far from satisfactory [26]. This study aimed to identify a novel prognostic biomarker for LUAD patients and help to guide individualized treatments and optimize the therapeutic strategy.

Several studies have demonstrated that SEC61G was overexpressed in various malignancies. However, the potential correlation between SEC61G and lung adenocarcinoma remains unclear. Our study showed that SEC61G was upregulated in LUAD tissues compared with adjacent normal tissues in the TCGA-LUAD cohort, which was consistent with previous studies founded in glioblastoma and hepatocellular carcinoma [14, 17]. Our analysis also found that SEC61G is significantly overexpressed in multiple malignancies in the TCGA database, such as gastric, liver cancer and breast carcinomas. These findings indicated that SEC61G could be a potential diagnostic marker in some type of cancers. In our study, the SEC61G gene was demonstrated as a potential diagnostic marker in LUAD, and its AUC exceeded 0.85. In the meanwhile, we also found that SEC61G correlated with the T stage, N stage and pathological grade of LUAD, further corroborating that the expression of SEC61G might be associated with the degree of malignancy of LUAD.

Then we demonstrated that patients with high SEC61G expression were significantly related to shorter overall survival (OS). Multivariate Cox regression analyses revealed that SEC61G expression served as an independent prognostic factor of OS. This result was also validated in LUAD patients from GEO database. Considering that SEC61G is a strong prognostic factor, we constructed a nomogram based on age, pathological stage and the SEC61G expression to predict the 1-, 3-and 5-year survival probability in LUAD patients. Calibration curves suggested that the actual prognosis was closely corresponded to the 1-and 3-year predicted prognosis, indicating good prediction performance of the nomogram. The nomogram could help to identify the high-risk patients and choose the more aggressive therapeutic strategy, which had better predictability than individual prognostic factors.

Although various mechanisms can contribute to elevated gene expression levels, DNA methylation and CNVs are two of the most common situations. DNA methylation can not only regulate gene expression but also plays a key role in tumorigenesis [27]. Previous studies demonstrated that several highly-expressed genes due to hypomethylation were associated with poor prognosis in NSCLC [28, 29]. Our results showed that SEC61G expression was associated with SEC61G hypomethylation (R = − 0.290, *p* < 0.001) and the promoter methylation level of SEC61G in LUAD is lower than that in normal tissue. More importantly, SEC61G methylation was related to the prognosis of LUAD, and hypomethylated patients have worse OS, which is in line with the prognostic value of the mRNA expression of SEC61G. Hence, SEC61G hypomethylation might partly contribute to SEC61G overexpression and was associated with poor prognosis in LUAD.

SEC61G encodes the core subunit of SEC61 complex in the endoplasmic reticulum membrane and plays a critical role in protein translocation and cellular calcium homeostasis [8]. Currently, the biological mechanism of SEC61G in tumors is still under exploration. In this study, GO and KEGG analysis indicated that SEC61G was significantly associated with the proliferation-associated biological process such as DNA replication, cell cycle and cell division, which is consistent with previous research in hepatocellular carcinoma [14]. To further verify the biological mechanism of SEC61G, we conducted a series of In vitro experiments in A427 and H1299 cells. Our experiments here demonstrated that knockdown of SEC61G inhibits LAC cell proliferation, invasion, and favorited apoptosis, which is in accordance with our bioinformatic prediction. It is worth noting that GSEA analysis found SEC61G was significantly related to the E2F targets pathway. E2Fs are a complex family of transcriptional regulators and play a key role in protecting cells from cell cycle-generated genomic errors and abnormal proliferation [30]. Li et al. found that overexpressed E2F genes were associated with poor prognosis in lung cancer patients [31]. Therefore, SEC61G might participate in the E2F-related pathway to regulate the cell cycle of lung cancer cells. Based on the above findings, overexpression of SEC61G might take an active part in cell cycle, cell division and E2F-related pathway in LUAD and leading to the occurrence and progression of lung cancer.

Another important finding was that SEC61G was significantly related to the unfolded protein response (UPR), which is an adaptive mechanism to reinstate ER proteostasis under the ER stress. Potent ER stress responses have been reported in the majority of human cancer, including brain, lung, breast, colon, gastric, pancreatic, prostate skin. Productive, non-lethal ER stress or activation of UPR-mediated cytoprotective functions, could promote tumorigenesis, mediate resistance to treatment and orchestrate various immune-evasive mechanisms [32]. UPR modulators such as IRE1α kinase inhibitors, PERK inhibitors and eIF2α inhibitors have shown notable anti-tumor efficacy in preclinical cancer models and hence provide a new insight of targets therapies [33–35]. It’s worth noting that SEC61G might confers a selective growth advantage by facilitating a cytoprotective response to ER stress [17]. Given that Our study showed significant association between SEC61G expression and ER stress response, we suspected that high expression of SEC61G play a critical role in malignant tumor behavior of LUAD via adaptive ER stress responses and SEC61G-related inhibitors might encouraging for the potential treatment of LUAD. Further studies are needed to explore the detailed molecular mechanisms through which ER stress response pathways stimulates the expression of SEC61G and induce its prosurvival effects. With the advent of immunotherapy, the tumor microenvironment has received more and more attention. Studies have shown that immune cells account for a large proportion of the tumor microenvironment and play a critical role in tumor development [25]. Our study demonstrated that the SEC61G expression was negatively correlated with CD4+ T cells, CD8+ T cells, NK cells, DCs and B cells, indicating that SEC61G might play an inhibitory role in both innate immunity and adaptive immunity [36]. There is accumulating evidence indicating that high levels of CD4+ T cells, CD8+ T cells, NK cells, DCs, and B cells infiltration are correlated with better prognosis in LUAD patients [37–39]. Additionally, patients with higher levels of CD8+ T cells and CD4+ cells infiltration were more likely to benefit from immunotherapy [40, 41]. Interestingly, We found that metastatic urothelial cancer patients with higher SEC61G expression level had a worse prognosis than those patients with low SEC61G expression who both received the atezolizumab immunotherapy in The IMvigor 210 study. Hence, it is reasonable to speculate that the SEC61G-mediatedimmunosuppression was one of the underlying causes of poor outcomes in LUAD patients and could provide a reference for the efficacy of immunotherapy in LUAD patients.

Although this current study enhanced a better understanding of the relationship between SEC61G and LUAD, some limitations of our study needed to be noted. First, our investigations into the role of SEC61G in tumors were based on the TIMER, TCGA and GEO databases, which lacks verification from our own clinical samples. Second, given that our study design is limited, additional key signaling pathways associated with SEC61G might be missed, and specific details on these relevant pathways and SEC61G-mediated immunosuppression are still unclear. Traditional in-house experimental studies and prospective studies are needed for further validation.

## Conclusion

In summary, using bioinformatic analysis, we systematically analyzed the expression pattern and prognostic value of SEC61G in LUAD patients from various databases. Our results indicated that high SEC61G expression correlated with worse prognosis and SEC61G was an independent prognostic factor for overall survival for LUAD patients. Additionally, vitro experiments verified the biological behaviors of SEC61G in lung cancer. Large-scale and comprehensive researches are needed to strengthen the findings before promoting the clinical efficacy of SEC61G as a prognostic biomarker and therapeutic target.

## Supplementary Information

**Additional file 1.**

**Additional file 2.**

**Additional file 3.**

## Data Availability

The datasets used and/or analyzed during the current study are available from the corresponding author on reasonable request.

## Abbreviations

SEC61G: Sec61 gamma subunit
LUAD: Lung adenocarcinoma
LAC: Lung adenocarcinoma cell
NSCLC: Non-small cell lung cancer
ER: Endoplasmic reticulum
UPR: Unfolded protein response
TCGA: The Cancer Genome Atlas
GEO: Gene Expression Omnibus
GSEA: Gene set enrichment analysis
GO: Gene ontology
KEGG: Kyoto Encyclopedia of Genes and Genomes
CNV: Copy number variation
FBS: Foetal bovine serum
siRNAs: Small interfering RNAs
NC: Negative control
OS: Overall survival

## References

[1] Siegel RL, Miller KD, Jemal A (2020). Cancer statistics, 2020. CA Cancer J Clin.

[2] Chen Z, Fillmore CM, Hammerman PS, Kim CF, Wong KK (2014). Non-small-cell lung cancers: a heterogeneous set of diseases. Nat Rev Cancer.

[3] Molina JR, Yang P, Cassivi SD, Schild SE, Adjei AA (2008). Non-small cell lung cancer: epidemiology, risk factors, treatment, and survivorship. Mayo Clin Proc.

[4] Allemani C, Matsuda T, Di Carlo V, Harewood R, Matz M, Nikšić M, Bonaventure A, Valkov M, Johnson CJ, Estève J (2018). Global surveillance of trends in cancer survival 2000–14 (CONCORD-3): analysis of individual records for 37 513 025 patients diagnosed with one of 18 cancers from 322 population-based registries in 71 countries. Lancet.

[5] Stella GM, Luisetti M, Pozzi E, Comoglio PM (2013). Oncogenes in non-small-cell lung cancer: emerging connections and novel therapeutic dynamics. Lancet Respir Med.

[6] Nanavaty P, Alvarez MS, Alberts WM (2014). Lung cancer screening: advantages, controversies, and applications. Cancer Control.

[7] Greenfield JJ, High S (1999). The Sec61 complex is located in both the ER and the ER-Golgi intermediate compartment. J Cell Sci.

[8] Linxweiler M, Schick B, Zimmermann R (2017). Let's talk about secs: Sec61, Sec62 and Sec63 in signal transduction, oncology and personalized medicine. Signal Transduct Target Ther.

[9] Liu Y, Ji W, Shergalis A, Xu J, Delaney AM, Calcaterra A, Pal A, Ljungman M, Neamati N, Rehemtulla A (2019). Activation of the unfolded protein response via inhibition of protein disulfide isomerase decreases the capacity for DNA repair to sensitize glioblastoma to radiotherapy. Cancer Res.

[10] Casper M, Weber SN, Kloor M, Mullenbach R, Grobholz R, Lammert F, Zimmer V (2013). Hepatocellular carcinoma as extracolonic manifestation of lynch syndrome indicates SEC63 as potential target gene in hepatocarcinogenesis. Scand J Gastroenterol.

[11] Wemmert S, Lindner Y, Linxweiler J, Wagenpfeil S, Bohle R, Niewald M, Schick B (2016). Initial evidence for Sec62 as a prognostic marker in advanced head and neck squamous cell carcinoma. Oncol Lett.

[12] Liu B, Liu J, Liao Y, Jin C, Zhang Z, Zhao J, Liu K, Huang H, Cao H, Cheng Q (2019). Identification of SEC61G as a novel prognostic marker for predicting survival and response to therapies in patients with glioblastoma. Med Sci Monit.

[13] Tsukamoto Y, Uchida T, Karnan S, Noguchi T, Nguyen LT, Tanigawa M, Takeuchi I, Matsuura K, Hijiya N, Nakada C, Kishida T, Kawahara K, Ito H, Murakami K, Fujioka T, Seto M, Moriyama M (2008). Genome-wide analysis of DNA copy number alterations and gene expression in gastric cancer. J Pathol.

[14] Gao H, Niu W, He Z, Gao C, Peng C, Niu J (2020). SEC61G plays an oncogenic role in hepatocellular carcinoma cells. Cell Cycle.

[15] Li WT, Zou AE, Honda CO, Zheng H, Wang XQ, Kisseleva T, Chang EY, Ongkeko WM (2019). Etiology-Specific Analysis of Hepatocellular Carcinoma Transcriptome Reveals Genetic Dysregulation in Pathways Implicated in Immunotherapy Efficacy. Cancers (Basel).

[16] Reis-Filho JS, Pinheiro C, Lambros MB, Milanezi F, Carvalho S, Savage K, Simpson PT, Jones C, Swift S, Mackay A, Reis RM, Hornick JL, Pereira EM, Baltazar F, Fletcher CDM, Ashworth A, Lakhani SR, Schmitt FC (2006). EGFR amplification and lack of activating mutations in metaplastic breast carcinomas. J Pathol.

[17] Lu Z, Zhou L, Killela P, Rasheed AB, Di C, Poe WE, McLendon RE, Bigner DD, Nicchitta C, Yan H (2009). Glioblastoma proto-oncogene SEC61gamma is required for tumor cell survival and response to endoplasmic reticulum stress. Cancer Res.

[18] Li T, Fan J, Wang B, Traugh N, Chen Q, Liu JS, Li B, Liu XS (2017). TIMER: a web server for comprehensive analysis of tumor-infiltrating immune cells. Cancer Res.

[19] Barretina J, Caponigro G, Stransky N, Venkatesan K, Margolin AA, Kim S, Wilson CJ, Lehar J, Kryukov GV, Sonkin D (2012). The Cancer cell line encyclopedia enables predictive modelling of anticancer drug sensitivity. Nature.

[20] Mariathasan S, Turley SJ, Nickles D, Castiglioni A, Yuen K, Wang Y, Kadel EE, Koeppen H, Astarita JL, Cubas R (2018). TGFbeta attenuates tumour response to PD-L1 blockade by contributing to exclusion of T cells. Nature.

[21] Zhou Y, Zhou B, Pache L, Chang M, Khodabakhshi AH, Tanaseichuk O, Benner C, Chanda SK (2019). Metascape provides a biologist-oriented resource for the analysis of systems-level datasets. Nat Commun.

[22] Subramanian A, Tamayo P, Mootha VK, Mukherjee S, Ebert BL, Gillette MA, Paulovich A, Pomeroy SL, Golub TR, Lander ES, Mesirov JP (2005). Gene set enrichment analysis: a knowledge-based approach for interpreting genome-wide expression profiles. Proc Natl Acad Sci U S A.

[23] Yu G, Wang LG, Han Y (2012). He QY: clusterProfiler: an R package for comparing biological themes among gene clusters. OMICS.

[24] Hanzelmann S, Castelo R, Guinney J (2013). GSVA: gene set variation analysis for microarray and RNA-seq data. BMC Bioinformatics.

[25] Bindea G, Mlecnik B, Tosolini M, Kirilovsky A, Waldner M, Obenauf AC, Angell H, Fredriksen T, Lafontaine L, Berger A, Bruneval P, Fridman WH, Becker C, Pagès F, Speicher MR, Trajanoski Z, Galon J (2013). Spatiotemporal dynamics of intratumoral immune cells reveal the immune landscape in human cancer. Immunity.

[26] Gettinger SN, Horn L, Gandhi L, Spigel DR, Antonia SJ, Rizvi NA, Powderly JD, Heist RS, Carvajal RD, Jackman DM, Sequist LV, Smith DC, Leming P, Carbone DP, Pinder-Schenck MC, Topalian SL, Hodi FS, Sosman JA, Sznol M, McDermott DF, Pardoll DM, Sankar V, Ahlers CM, Salvati M, Wigginton JM, Hellmann MD, Kollia GD, Gupta AK, Brahmer JR (2015). Overall survival and long-term safety of Nivolumab (anti-programmed death 1 antibody, BMS-936558, ONO-4538) in patients with previously treated advanced non-small-cell lung Cancer. J Clin Oncol.

[27] Klutstein M, Nejman D, Greenfield R, Cedar H (2016). DNA methylation in Cancer and aging. Cancer Res.

[28] Sato T, Soejima K, Arai E, Hamamoto J, Yasuda H, Arai D, Ishioka K, Ohgino K, Naoki K, Kohno T (2015). Prognostic implication of PTPRH hypomethylation in non-small cell lung cancer. Oncol Rep.

[29] Noguera-Ucles JF, Boyero L, Salinas A, Cordero Varela JA, Benedetti JC, Bernabe-Caro R, Sanchez-Gastaldo A, Alonso M, Paz-Ares L, Molina-Pinelo S (2020). The Roles of Imprinted SLC22A18 and SLC22A18AS Gene Overexpression Caused by Promoter CpG Island Hypomethylation as Diagnostic and Prognostic Biomarkers for Non-Small Cell Lung Cancer Patients. Cancers (Basel).

[30] Kent LN, Leone G (2019). The broken cycle: E2F dysfunction in cancer. Nat Rev Cancer.

[31] Sun CC, Zhou Q, Hu W, Li SJ, Zhang F, Chen ZL, Li G, Bi ZY, Bi YY, Gong FY, Bo T, Yuan ZP, Hu WD, Zhan BT, Zhang Q, Tang QZ, Li DJ (2018). Transcriptional E2F1/2/5/8 as potential targets and transcriptional E2F3/6/7 as new biomarkers for the prognosis of human lung carcinoma. Aging (Albany NY).

[32] Chen X, Cubillos-Ruiz JR (2021). Endoplasmic reticulum stress signals in the tumour and its microenvironment. Nat Rev Cancer.

[33] Harnoss JM, Le Thomas A, Shemorry A, Marsters SA, Lawrence DA, Lu M, Chen YA, Qing J, Totpal K, Kan D (2019). Disruption of IRE1alpha through its kinase domain attenuates multiple myeloma. Proc Natl Acad Sci U S A.

[34] Hetz C, Axten JM, Patterson JB (2019). Pharmacological targeting of the unfolded protein response for disease intervention. Nat Chem Biol.

[35] Rabouw HH, Langereis MA, Anand AA, Visser LJ, de Groot RJ, Walter P, van Kuppeveld FJM (2019). Small molecule ISRIB suppresses the integrated stress response within a defined window of activation. Proc Natl Acad Sci U S A.

[36] Gajewski TF, Schreiber H, Fu YX (2013). Innate and adaptive immune cells in the tumor microenvironment. Nat Immunol.

[37] Aktas ON, Ozturk AB, Erman B, Erus S, Tanju S, Dilege S (2018). Role of natural killer cells in lung cancer. J Cancer Res Clin Oncol.

[38] Wang SS, Liu W, Ly D, Xu H, Qu L, Zhang L (2019). Tumor-infiltrating B cells: their role and application in anti-tumor immunity in lung cancer. Cell Mol Immunol.

[39] Remark R, Becker C, Gomez JE, Damotte D, Dieu-Nosjean MC, Sautes-Fridman C, Fridman WH, Powell CA, Altorki NK, Merad M (2015). The non-small cell lung cancer immune contexture. A major determinant of tumor characteristics and patient outcome. Am J Respir Crit Care Med.

[40] Doroshow DB, Sanmamed MF, Hastings K, Politi K, Rimm DL, Chen L, Melero I, Schalper KA, Herbst RS (2019). Immunotherapy in non-small cell lung Cancer: facts and hopes. Clin Cancer Res.

[41] Hurkmans DP, Kuipers ME, Smit J, van Marion R, Mathijssen RHJ, Postmus PE, Hiemstra PS, Aerts J, von der Thusen JH, van der Burg SH (2020). Tumor mutational load, CD8(+) T cells, expression of PD-L1 and HLA class I to guide immunotherapy decisions in NSCLC patients. Cancer Immunol Immunother.

