# Supplementary figures and images for: Prognostic value of SEC61G in lung adenocarcinoma: a comprehensive study based on bioinformatics and in vitro validation
Source: BMC Cancer. 2021 Nov 13;21:1216. doi: 10.1186/s12885-021-08957-4 (PMC8590767; doi:10.1186/s12885-021-08957-4)

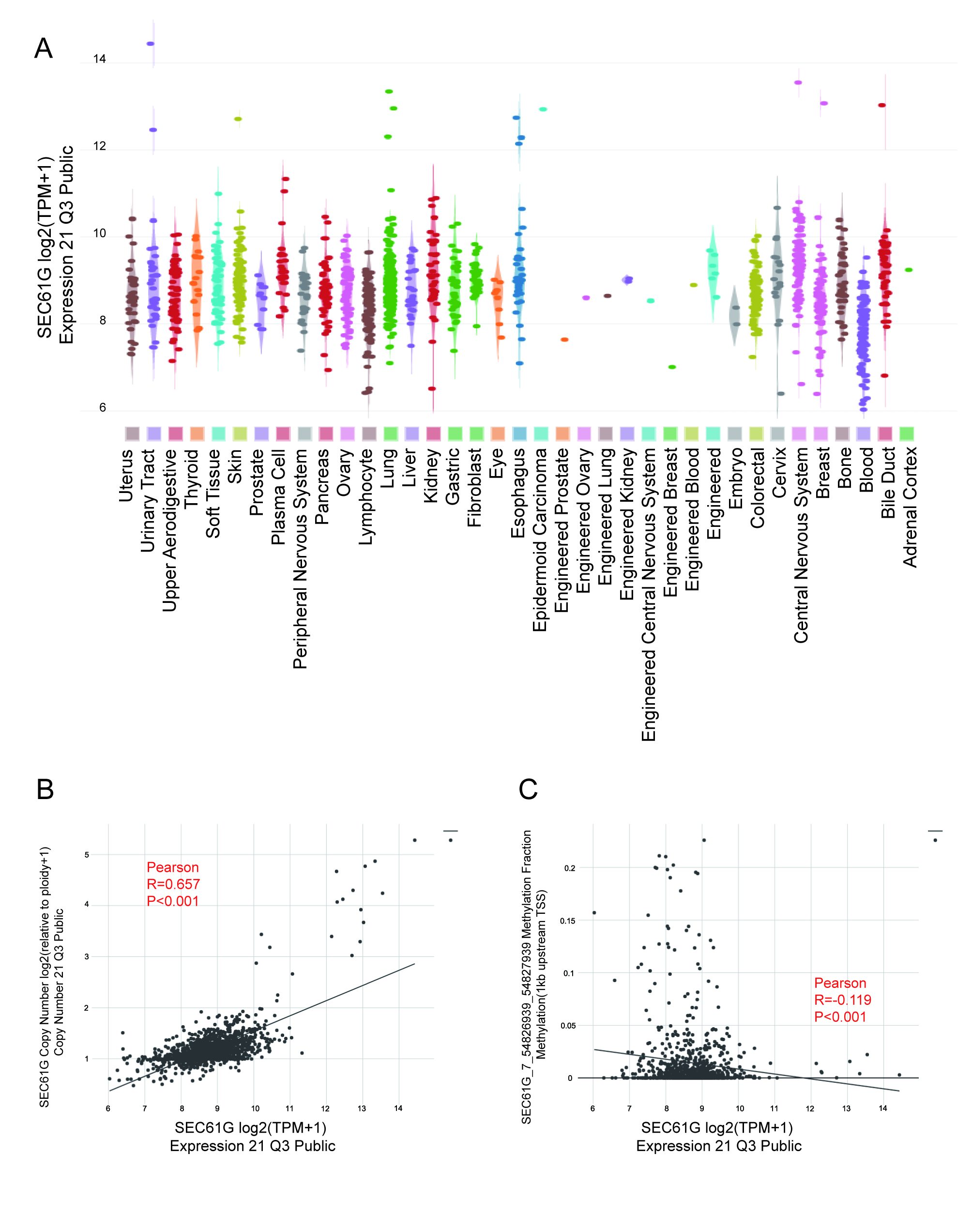

Supplement: Supplementary file 1 — Additional file 1. [file 12885_2021_8957_MOESM1_ESM.tif]

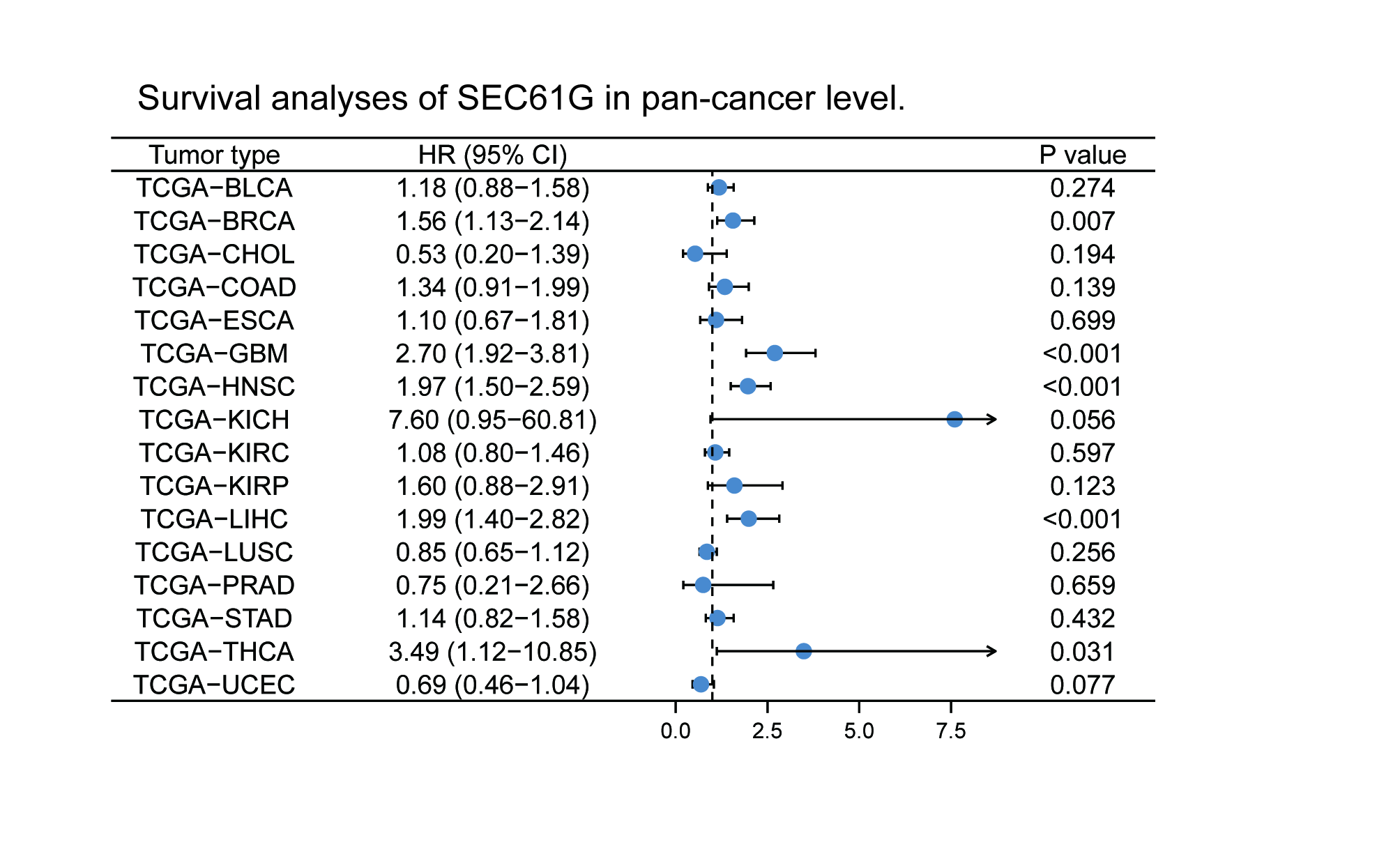

Supplement: Supplementary file 2 — Additional file 2. [file 12885_2021_8957_MOESM2_ESM.tif]

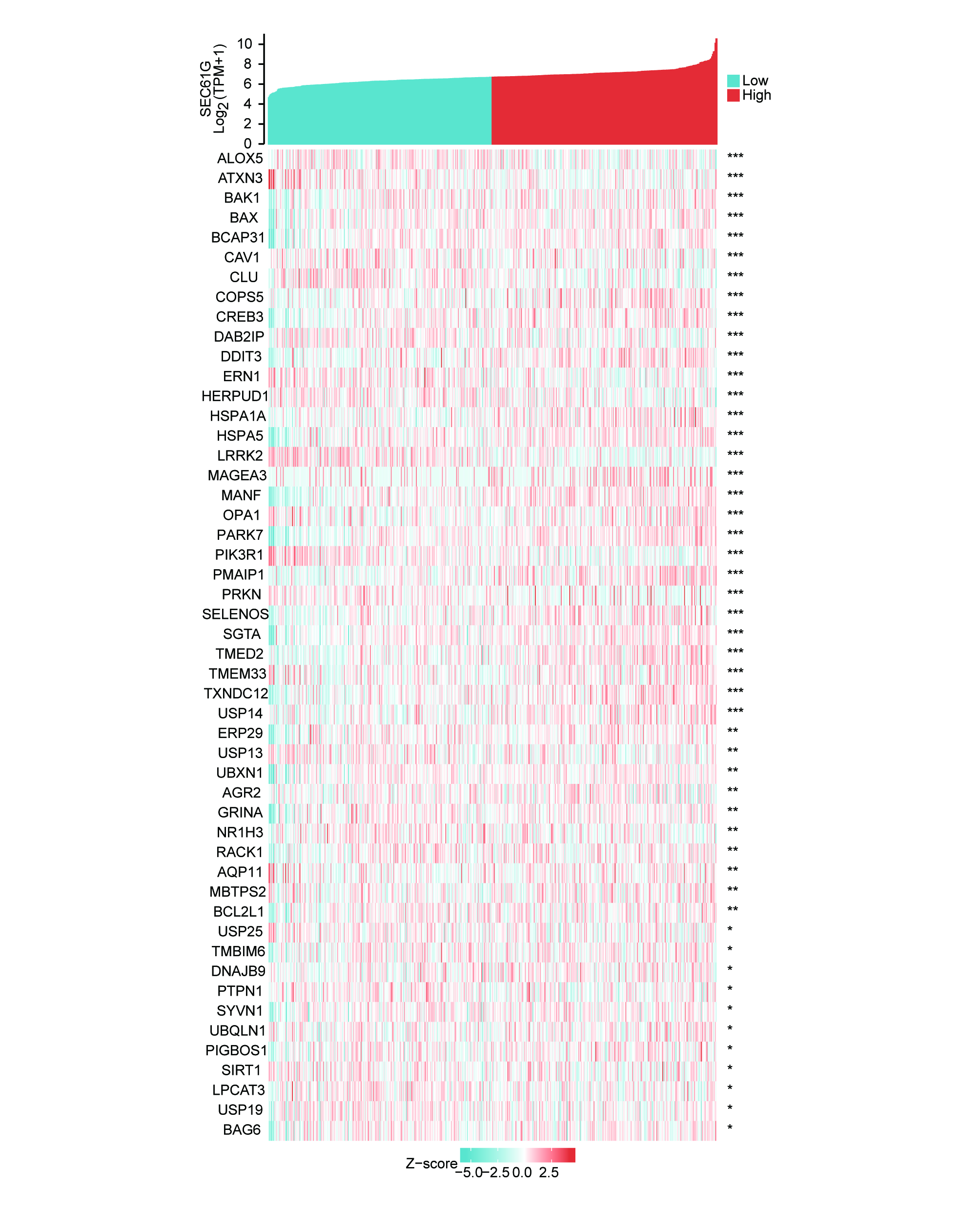

Supplement: Supplementary file 3 — Additional file 3. [file 12885_2021_8957_MOESM3_ESM.tif]
